# Effectiveness of preoperative beta-blockade on intra-operative heart rate in vascular surgery cases conducted under regional or local anesthesia

**DOI:** 10.1186/2193-1801-3-227

**Published:** 2014-05-05

**Authors:** Seshadri C Mudumbai, Todd Wagner, Satish Mahajan, Robert King, Paul A Heidenreich, Mark Hlatky, Arthur W Wallace, Edward R Mariano

**Affiliations:** Anesthesiology and Perioperative Care Service, Veterans Affairs Palo Alto Health Care System, 3801 Miranda Avenue, Palo Alto, CA 94304 USA; Department of Anesthesiology, Perioperative, and Pain Medicine, Stanford University School of Medicine, Stanford, USA; Center for Innovation to Implementation, Veterans Affairs Palo Alto Health Care System, Palo Alto, USA; Department of Nursing, Veterans Affairs Palo Alto Health Care System, Palo Alto, USA; Cardiology Service, Veterans Affairs Palo Alto Health Care System, Palo Alto, USA; Department of Cardiology, Stanford University School of Medicine, Stanford, USA; Department of Health Research and Policy and Department of Medicine (Cardiovascular Medicine), Stanford, USA; Anesthesia Service, San Francisco Veterans Affairs Medical Center, San Francisco, CA USA; Department of Anesthesiology and Perioperative Care, University of California San Francisco, San Francisco, CA USA

**Keywords:** Perioperative medicine, Vascular surgery, β-blockers, Heart rate, General anesthesia, Regional anesthesia, Monitored anesthesia care, Effectiveness

## Abstract

**Background:**

Preoperative β-blockade has been posited to result in better outcomes for vascular surgery patients by attenuating acute hemodynamic changes associated with stress. However, the incremental effectiveness, if any, of β-blocker usage in blunting heart rate responsiveness for vascular surgery patients *who avoid general anesthesia* remains unknown.

**Methods:**

We reviewed an existing database and identified 213 consecutive vascular surgery cases from 2005–2011 conducted without general anesthesia (i.e., under monitored anesthesia care or regional anesthesia) at a tertiary care Veterans Administration medical center and categorized patients based on presence or absence of preoperative β-blocker prescription. For this series of patients, with the primary outcome of maximum heart rate during the interval between operating room entry to surgical incision, we examined the association of maximal heart rate and preoperative β-blocker usage by performing crude and multivariate linear regression, adjusting for relevant patient factors.

**Results:**

Of 213 eligible cases, 137 were prescribed preoperative β*-*blockers, and 76 were not. The two groups were comparable across baseline patient factors and intraoperative medication doses. The β-blocker group experienced lower maximal heart rates during the period of evaluation compared to the non-β-blocker group (85 ± 22 bpm vs. 98 ± 36 bpm, respectively; p = 0.002). Adjusted linear regression confirmed a statistically-significant association between lower maximal heart rate and the use of β-blockers (Beta = -11.5; 95% CI [-3.7, -19.3] p = 0.004).

**Conclusions:**

The addition of preoperative β-blockers, even when general anesthesia is avoided, may be beneficial in further attenuating stress-induced hemodynamic changes for vascular surgery patients.

## Introduction

Vascular surgery patients continue to be a high risk population for perioperative cardiac morbidity and mortality (Fleisher et al. 2007). When managing these patients during the vulnerable period surrounding surgery, both β-blockers and avoidance of general anesthesia (i.e., choosing local or regional anesthesia alternatives) have been posited to result in better outcomes (Fleisher et al. 2009; Wu and Fleisher 2000). By improving myocardial oxygen balance, stabilizing coronary plaques, and increasing thresholds for developing fatal arrhythmias, β-blocker therapy may help to decrease adverse cardiac events for patients with underlying coronary artery disease and/or rate-related ischemia (López-Sendó et al. 2004). By avoiding general anesthesia, regional anesthesia or monitored anesthesia care (MAC) with local anesthesia techniques may prevent acute hemodynamic changes such as tachycardia commonly observed during anesthetic induction with airway instrumentation (Moraca et al. 2003; Beattie et al. 2001; Parker et al. 2000; Rerkasem and Rothwell 2008). These brief but intense episodes of tachycardia are of particular concern for patients with severe congestive heart failure or pulmonary hypertension, which are not uncommon comorbidities in vascular surgery patients (Beattie et al. 2008).

Our group has previously demonstrated that for patients undergoing general anesthesia for vascular surgery, preoperative β-blockade is associated with decreased maximal heart rates for the period prior to surgical incision (Mudumbai et al. 2012). The added benefit, if any, of preoperative β-blockade *in addition to avoiding general anesthesia* in attenuating heart rate responsiveness during surgery has not been previously investigated. We hypothesized that vascular surgery patients taking β-blockers preoperatively and who avoid general anesthesia will experience lower maximal heart rates during the intraoperative period prior to surgical incision compared with those not taking β-blockers.

## Subjects and methods

With institutional review board approval and waiver of informed consent from our affiliated university, we created a database of adult patients who successively underwent elective or emergent vascular surgery from 2005–2011 at a tertiary care Veterans Administration (VA) medical center using the VA integrated electronic health record, VISTA, International Classification of Diseases (ICD)-9 diagnosis codes, and Current Procedural Terminology (CPT) codes. For the current study, only cases whose administrative data indicated the use of monitored anesthesia care or regional anesthesia were included; documentation of general anesthesia or presence of an inhaled anesthetic agent on the intraoperative anesthetic record represented criteria for exclusion. We aggregated each patient’s demographic and preoperative prescription information. Details of surgery such as key timepoints (i.e., entry to operating room, surgical incision), procedural duration, the type of anesthesia (e.g., local/MAC or regional), and type of procedure were extracted from the electronic health record and used to identify eligible cases. After chart review, we grouped patients based on their use of preoperative β-blockers; patients with a documented active oral β-blocker prescription for ≥ 1 month before surgery were included in the β-blocker group while all others were included in the no β-blocker group. The present study employed methods similar to a previous study although the cases selected represented a distinct and separate sample and did not overlap with the sample used for the previous study (Mudumbai et al. 2012).

### Outcomes

The primary outcome was maximal heart rate observed during the interval between entry to the operating room (OR) and surgical incision, which we defined as the pre-surgical incision (PSI) period. Secondary outcomes included doses of intraoperative β-blockers, doses of other medications (e.g., sedatives, opioids, vasopressors), and hemodynamic variables (e.g., heart rate, systolic and diastolic blood pressure) and cardiac rhythm during the PSI period and throughout the entire operation. We also evaluated incident major adverse cardiac event, stroke, and all-cause 30-day mortality for each group. Intraoperative hemodynamic and medication data for each patient were collected from our institution’s anesthesia information management system (AIMS) database (PICIS 7.2 and 8.1; Picis Inc, Wakefield, MA, USA) and linked to the patient’s other data using social security number as a key.

### Statistical analysis

Normality of distribution was evaluated using the Kolmogorov-Smirnov test. Categorical variables were compared using the chi-square test or Fisher’s exact test when appropriate (n < 5 in any field). For continuous variables, we used Student’s *t*-test for normally-distributed data or the Wilcoxon rank-sum test for non-normal distributions. We conducted crude and adjusted ordinary least squares adjusted linear regression to evaluate the potential association between maximal heart rate during the PSI period and preoperative β-blocker use. The following factors were included as potential confounders a priori for the adjusted linear regression: age, body mass index, and medications given during the PSI period (e.g., opioids, sedatives, propofol or etomidate, vasopressors, intraoperative β-blocker). With the primary outcome not normally distributed, we constructed a model to test the association between the logarithm of maximal heart rate and preoperative β-blocker use with adjustment for patient factors. To account for skewed distributions and dose-dependent non-linear effects of medications given in the PSI period on heart rate, a third model included these medication dosages converted into categorical variables based on tertiles (i.e., low, moderate, and high dose). We also compared the number of patients who were administered additional β-blockade between our two groups. Statistical analyses were performed using Stata 12.1 (Stata Corporation, College Station, TX, USA) with a two-sided p ≤ 0.05 considered statistically-significant.

## Results

**Figure 1:**
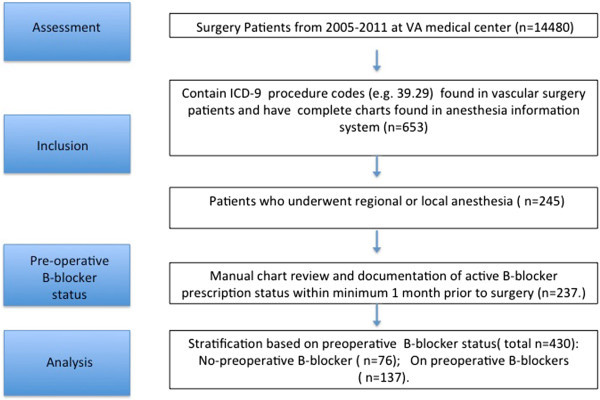
**Selection of patients.**

Two hundred thirteen cases were eligible for analysis (Figure 1). Table 1 presents patient demographic, morphometric information, surgical measures, and medication doses for each group based on preoperative β-blocker usage. Prescriptions for preoperative β-blockers were identified for 64% of patients. Table 2 presents surgical procedures and anesthetic techniques for both groups; approximately 75% of our cases were conducted under MAC.

**Table 1:** **Patient, surgical characteristics, and medications for pre-surgical incision period**

Patient characteristics	No preoperative β-blocker (n = 76)	Preoperative β-blocker (n = 137)	P-value
Age (yrs)	68.2 (11.8)	65.7 (10.2)	0.11
Weight (kg)	84.8 (17.0)	84.4 (18.3)	0.87
Height (cm)	175.0 (9.5)	173.4 (8.1)	0.22
BMI (kg/m^2^)	27.7 (4.9)	28.0 (5.5)	0.66
ASA Status (mode)	III	III	-
Sex (male/female)	76/0	137/0	-
Duration for pre surgical incision period (mins)	70.6 (21.9)	59.1 (28.0)	0.007
Duration for entire surgical case (mins)	195.2 (104.4)	163.4 (77.6)	0.016
**Pre-surgical incision medications**			
Fentanyl equivalents (mcg)	90 (158)	54 (64)	0.02
Midazolam (mg)	2 (1.4)	1.7 (0.6)	0.02
Propofol (mg)	51.8 (60)	110 (94)	0.21
Etomidate (mg)	15.7 (6.3)	17.2 (5.5)	0.69
Labetalol(mg)	5 (0)*	35 (28.2)	< 0.001
Metoprolol (mg)	3 (0)*	2.7 (2.1)	< 0.001
Esmolol (mg)	**	30 (0)*	-
Ephedrine (mg)	10 (7.7)	9.5 (4.9)	0.87
Phenylephrine (mcg)	258 (142)	160 (69)	0.30

Data are presented as mean (SD) or number of subjects when applicable. *One patient received this medication. **No patients in this group received esmolol.

**Table 2:** **Vascular surgical procedures and anesthetic techniques**

		No preoperative β-blocker (n = 76)	Preoperative β-blocker (n = 137)	P-value
	Arterio-venous grafts	41	102	<0.01
	Peripheral Revascularizations (e.g., femoral-popliteal bypass)	7	12	0.79
	Endovascular Abdominal Aortic Aneurysm Repair	9	5	0.04
**Procedures**	Wound Exploration and Debridements	3	3	0.66
	Amputations	12	8	0.03
	*Above Knee*	2	2	0.62
	*Below Knee*	8	4	0.03
	*Foot*	2	2	0.62
	Carotid Endarterectomy	4	6	0.74
	IVC filter	1	1	1.00
**Anesthetic techniques**	Regional	Total = 22	Total = 31	0.32
	*Neuraxial*	16	17	0.11
	*Peripheral Nerve Block*	6	14	0.63
	Monitored Anesthesia Care	54	106	0.32

Data are presented as number of subjects.

### Primary outcome

**Figure 2:**
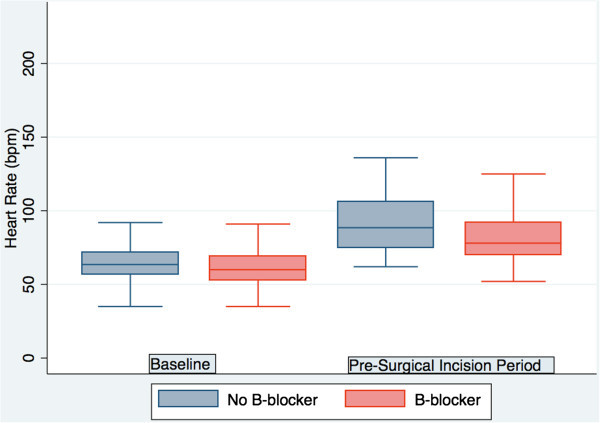
**Box-plots of heart rate at baseline and maximal heart rate during the pre-surgical incision period.** Baseline values represent data collected upon entry to the operating room; bpm = beats per minute; boxes represent the 25th-75th percentiles; whiskers represent 5-95th percentiles. The β-blocker group experienced lower maximal heart rates compared to the non-β-blocker group (85 ± 22 bpm vs. 98 ± 36 bpm, respectively; p = 0.002).

Maximal heart rate during the PSI period was lower for the β-blocker group compared to the non-β-blocker group (85 ± 22 bpm vs. 98 ± 36 bpm, respectively; p = 0.002, Figure 2). This statistically-significant association between a lower maximal heart rate and the use of β-blockers (Beta = -11.5; 95% CI [-3.7, -19.3]; p = 0.004) withstood adjustment for relevant patient factors. In the multivariate regression, beta-coefficients for propofol, etomidate, and phenylephrine in the linear regression were statistically-significant but low: propofol (Beta = 0.10; 95% CI [0.04, 0.16]; p = 0.001); etomidate (Beta = 0.52; 95% CI [0.07, 0.90]; p = 0.020); and phenylephrine (Beta = 0.02; 95% CI [0.004, 0.04]; p = 0.020).

The results from our initial model were confirmed by the two alternative regression analyses. First, when using the logarithm of maximal heart rate as an outcome, the largest effect was again seen with preoperative β-blocker use (Beta = -0.097; 95% CI [-0.15, -0.31]; p = 0.002), which was equivalent to a 10-bpm decrease in maximal heart rate. Second, a similar association between lower maximal heart rate and the use of β-blockers (Beta = -11.4; 95% CI [-18.2, -4.5]; p = 0.001) was observed after we converted our PSI period medication doses into low, medium, and high tertiles. A post-hoc calculation revealed a power of 89% to detect a difference of 13 bpm with the present study.

### Secondary outcomes

There was no difference between groups regarding intraoperative dosing of β-blockers or the number of patients who were given additional β-blockade (Table 3). The non-β-blocker group had lower systolic blood pressure on entry to the OR compared to the β-blocker group (125 ± 29 mmHg vs. 135 ± 30 mmHg, respectively; p = 0.036) but higher maximal heart rate during the entire operation (103 ± 37 vs. 88 ± 22, respectively, p < 0.001). There were no differences in other intraoperative hemodynamic parameters. The majority of patients in both groups, 113/137 β-blocker and 57/76 non-β-blocker, were in normal sinus rhythm for the PSI period (p = 0.210). Within 30 days after surgery, we found no new instances of major adverse cardiac event or stroke for either group; 2 patients died in the β-blocker group compared to 1 patient in the non-β-blocker group (Relative Risk = 0.93; 95% CI [0.19-4.70]).

**Table 3:** **Intra-operative β-blocker doses for entire surgical case**

Type of β-blocker	No preoperative β-blocker (n = 76)	Preoperative β-blocker (n = 137)	P-values
Labetalol (mg)	29.6 (39.6)	38.5 (47.3)	0.71
Metoprolol (mg)	6.7 (5.7)	6.0 (3.9)	0.68
Esmolol (mg)	35.0 (7.1)	70.0 (43.5)	0.36

Data are presented as mean (SD).

## Discussion

Preoperative β-blockade with local or regional anesthesia for vascular surgery is associated with lower maximal heart rate intraoperatively compared to local or regional anesthesia alone. These results support the findings of a previous study that demonstrated the effectiveness of β-blockade on heart rate control during the induction period for vascular surgery patients undergoing general anesthesia (Mudumbai et al. 2012). Unlike the previous study, all patients in the present study had average maximal heart rates below 100 bpm regardless of preoperative β-blocker status. Since heart rates over 100 bpm are associated with an increase in myocardial infarction, our study results suggest a potential advantage in avoiding general anesthesia when appropriate for the surgical procedure and that preoperative β-blockade in the perioperative management of high-risk vascular surgery patients may offer additional effects in terms of heart rate control (Beattie et al. 2008).

Although this topic continues to be debated, published recommendations based on randomized controlled trials suggest that vascular surgery patients represent a unique cohort in which the advantages of perioperative β-blockade may outweigh risks (Fleisher et al. 2009; López-Sendó et al. 2004). However, patterns of intraoperative heart rate and medication-induced control during stressful periods and any association with cardiac morbidity in high-risk patients are collectively an active area of research (Fleisher and Poldermans 2008). The present study is the first to examine the effectiveness of preoperative β-blockade during the intraoperative period in a cohort of actual vascular surgery patients undergoing regional or monitored anesthesia care under situations of routine clinical practice. Although the 10-bpm decrease in maximal heart rate in favor of the β-blocker group did not directly translate into overall morbidity and mortality benefits, published studies have shown that elevated heart rates in 10-bpm increments may be associated with increased risk of myocardial ischemia, troponin-T release, and long-term mortality (Feringa et al. 2006). We did note a higher relative risk for 30-day mortality in our β-blocker population-similar to those of the POISE trial (Group et al. 2008). Since our study was neither designed nor powered to study 30-day mortality, incident major adverse cardiac event or stroke, we do not draw any definitive conclusions about these findings.

### Preoperative β-blockade and intraoperative heart rate control

The 10-bpm difference during the PSI period in favor of the β-blocker group in this study is clinically relevant given recent recommendations that heart rate should be tightly controlled within a range of 60–80 bpm with β-blockers throughout the perioperative period. Although both groups’ average maximal heart rates were over 80 bpm, the β-blocker group maintained heart rates much closer to the target range during the PSI period and entire operation compared to the non-β-blocker group.

Despite the use of regional or monitored anesthesia care and avoidance of anesthetic induction and airway manipulation, the PSI period remains a vulnerable period of physiologic stress for vascular surgery patients. One source of stress and hemodynamic instability during the PSI period may be the administration of site-specific local anesthetic or regional block (Tuman et al. 1991; Christopherson et al. 1993; Christopherson et al. 1996). Since patients are awake or receiving monitored anesthesia care, they may exhibit increases in heart rate due to anxiety, positioning, needlestick stimulation, pain from the local anesthetic itself, pain from manipulation of the affected body part, or inadvertent intravascular injection of epinephrine-containing local anesthetic solutions. In addition, a significant percentage of our patients (54% in the control and 74% in the intervention group) underwent arterio-venous (AV) fistulae and grafts. There are important clinical and physiologic differences in the PSI period between AV procedures and other vascular surgery procedures (e.g., peripheral revascularization, amputation); these differences may also contribute to observed maximum heart rates. Regardless of the actual procedure they undergo, increases in heart rate for these high-risk patients may disrupt the fragile myocardial oxygen supply/demand balance, leading to myocardial ischemia or cardiac dysrythmias. The PIRAT study involving patients undergoing lower extremity vascular surgery under epidural or general anesthesia has demonstrated an association between rapid heart rate changes (> 20 bpm in 5 minutes) and intraoperative myocardial ischemia (Christopherson et al. 1996). Following surgical incision, these patients still experience physiologic perturbations related to the surgical procedure and arguably may be more hemodynamically responsive since they are not under general anesthesia. The results of the present study suggest β-blocker-associated heart rate control throughout the intraoperative period beyond the PSI interval. Future effectiveness studies are necessary to help refine existing protocols for appropriate medication selection and target heart rates.

### Patterns of intraoperative medication use

Prior to surgical incision, non-β-blocked patients receive higher doses of opioids and sedatives and lower doses of β-blockers than preoperatively β-blocked patients. We speculate that, during the PSI period, anesthesiologists are likely to interpret elevated heart rate for non-β-blocked patients as pain or anxiety. After surgical incision, intraoperative doses of β-blockers are the same between groups, suggesting that anesthesiologists are more likely to interpret any increase in heart rate as not pain-related when the surgery is performed under local or regional anesthesia and feel administering β-blockers to control heart rate. These hypotheses related to medication administration patterns by providers and perioperative heart rate control must be more rigorously studied (Freundlich and Kheterpal 2011; Kheterpal 2009; Mukherjee and Eagle 2003).

### Study limitations

Since this is a retrospective cohort study, we can only identify associations and not draw definitive conclusions regarding causality between preoperative β-blockade and heart rate control. However, we designed this study as an effectiveness study and took several steps to minimize bias (Freundlich and Kheterpal 2011; Iglehart 2009). By focusing on the PSI period, we attempted to measure the effects of preoperative β-blockade without the confounders of surgical stimuli and hemodynamic shifts from blood loss that can also affect heart rate. We attempted to control for the effects of other medications in our main regression model and sensitivity analysis. We acknowledge that anesthesiologists may have altered their perioperative management for known β-blocker patients, and we attempted to minimize this source of bias by including a consecutive series of surgical patients within a broad time frame including years before publication of the ACC-AHA recommendations (Fleisher et al. 2007; Fleisher et al. 2009). Another limitation of the present study is the use of prescription records for assignment to β-blocker or non-β-blocker groups rather than actual medication administration data (not available for outpatients); therefore, we employed intent-to-treat analysis to account for non-compliance and inadvertent cross-overs (Lachin 2000). The present study did not specifically study the inclusion of epinephrine in local anesthetic solutions, the effects of other cardiovascular medications and the potential interactions between these medications and β-blockers, so this represents another area for future investigation (Cleophas et al. 2007). Lastly, this study was performed at one tertiary care, university-affiliated VA medical center, so the results may not be applicable to every institution or practice. For example, the overrepresentation of male patients in our sample, while typical of VA hospitals, limits generalizability to females. However, the sample size and duration of study, conditions of routine clinical practice, and heterogeneity of providers support the external validity of our study within this demographic.

## Conclusion

In summary, in a cohort of “real-world” vascular surgery patients who avoided general anesthesia, preoperative β-blockade is associated with a decrease in maximal heart rate observed during the PSI period. A lowered heart rate may offer potential protection against rate-induced myocardial ischemia for high-risk patients. Future studies are necessary to evaluate perioperative hemodynamic and medication administration patterns, optimal medication selection, and potential effects on postoperative morbidity and mortality for a wider variety of surgical patients.

## Abbreviations

PSI: Pre-surgical period
MAC: Monitored anesthesia care
OR: Operating room
VA: Veterans Administration
AV: Arterio-venous.
